# Assessing transfusion need in patients with type A aortic dissection with multiplate aggregometry

**DOI:** 10.1371/journal.pone.0324477

**Published:** 2025-07-17

**Authors:** Biniam Melese Bekele, Sascha Ott, Benjamin O’Brien, Matteo Montagner, Volkmar Falk, Stephan Kurz

**Affiliations:** 1 Charité – Universitätsmedizin Berlin, corporate member of Freie Universität Berlin and Humboldt- Universität zu Berlin, Berlin, Germany; 2 Deutsches Herzzentrum der Charité – Medical Heart Center of Charité and German Heart Institute Berlin, Department of Cardiothoracic and Vascular Surgery, Berlin, Germany; 3 Deutsches Herzzentrum der Charité – Medical Heart Center of Charité and German Heart Institute Berlin, Department of Cardiac Anesthesiology and Intensive Care Medicine, Berlin, Germany; 4 DZHK (German Centre for Cardiovascular Research), Partner Site Berlin, Berlin, Germany; 5 DZHK (German Centre for Cardiovascular Research), Partner Site Berlin, Berlin, Germany; 6 Department of Health Science and Technology, Translational cardiovascular technologies, Institute of Translational Medicine, Zürich, Switzerland; Klinikum Region Hannover GmbH, GERMANY

## Abstract

**Background:**

Acute type A aortic dissection (ATAAD) represents a life-threatening surgical emergency. Use of antiplatelet drugs and platelet dysfunction exacerbate the already high rates of postoperative morbidity and mortality. The objective of this study was to determine the relationship between preoperative platelet function evaluated through Multiplate® aggregometry and transfusion requirement.

**Methods:**

This observational retrospective study included 180 patients who underwent emergency surgical repair of ATAAD over a two-year period (2019–2021). Platelet function was evaluated preoperatively using Multiplate® analyzer with three activators: adenosine diphosphate (ADP test), arachidonic acid (ASPI test) and thrombin receptor-activating peptide-6 (TRAP test). Primary outcome was the number of blood products transfused. In addition, the need for surgical re-exploration, length of hospital stay and 30-day mortality were compared.

**Results:**

Abnormal aggregation responses were observed in 89 (49.2%) patients using ASPI test, in 47 (26%) patients using ADP test, and in 113 (63.5%) patients using the TRAP test. Thirty-six (20%) patients exhibited an abnormal response to all three tests. Preoperative use of aspirin was documented in 35 (19.3%) patients, while clopidogrel use was documented in 7 (3.9%) patients. Patients with anormal ADP responses required more intraoperative red cell concentrates (3.4 ± 3.9 versus 2.1 ± 3.2, *p* = 0.039), intraoperative platelet concentrates (4.2 ± 2.9 versus 3.1 ± 2.4, *p* = 0.015) and total platelet concentrates (8.1 ± 7.8 versus 6.2 ± 8.1, p = 0.008). There were no significant differences in the need for surgical re-exploration, the total length of hospital stay, or 30-day mortality.

**Conclusions:**

Multiplate® aggregometry can be a useful tool for evaluating platelet dysfunction and assessing transfusion need in ATAAD patients, thereby aiding in the optimization of treatment strategies.

## Introduction

Acute type A aortic dissection (ATAAD) is a life-threatening surgical emergency associated with a high mortality rate of 0.5% per hour in the first 48 hours. [1] Postoperative bleeding has been identified as an independent risk factor of morbidity and mortality in this patient population. [2] Platelet dysfunction, often influenced by disease pathology, preoperative antiplatelet drugs and comorbidities, significantly contributes to postoperative bleeding and subsequent complications. [3,4] ATAAD is associated with inflammatory reactions and platelet activation. This response is likely induced by the tear of the aortic wall and is closely correlated with the extent of dissection. [5] Furthermore, studies have shown that aortic dissection is often misdiagnosed as acute coronary syndrome, leading to the consecutive administration of antiplatelet drugs. [6,7] This, in turn, can increase the risk of bleeding in patients undergoing emergency surgical repair. [8]

The identification of patients at high risk of intra- and postoperative bleeding and the development of a reliable detection tool have been a focus of study for several decades. Studies using standard perioperative coagulation tests showed variable outcomes with marginal improvements in the prediction of bleeding. [9,10] However, Multiplate® aggregometry (MPA) has proven to be a reliable tool in different non-cardiac patient populations including trauma and neurosurgical patients. [11–13] MPA analyzes platelet function by assessing platelet aggregation in response to different agonists. The use of MPA as point-of-care (POC) diagnostics in cardiac surgery has recently gained traction, with studies showing benefits in predicting bleeding complications. [14,15] However, its application in ATAAD remains largely unexplored.

The aim of this study was to investigate the association between preoperative platelet function, evaluated through MPA, and postoperative bleeding and requirements of blood product transfusions.

## Methods

### Patient population

This observational study included patients with ATAAD who presented to a single high-volume cardiac center within a two-year period (2019–2021). Inclusion criteria were patients aged > 18 years, diagnosed with ATAAD, and surgically treated. Patients with iatrogenic aortic dissection were excluded. The study was conducted in accordance with the Declaration of Helsinki. The study was approved by the institutional ethics Committee (EA2/126/14) and waived the requirement for informed consent, due to the retrospective nature of the study and the collection and analysis of completely anonymized data. The retrospective data was accessed the latest on 11/02/2023.

### Multiplate aggregometry

Platelet function was assessed using multiple electrode platelet aggregometry (Multiplate® analyzer; Roche Diagnostics, Mannheim, Germany) with whole hirudin-containing blood samples, as described previously. [12] Platelet aggregation was measured in response to stimulation with arachidonic acid (ASPI-test, designed to evaluate the effect of aspirin), adenosine diphosphate (ADP-test, designed to evaluate the effect of P2Y12 antagonists, thienopyridines and non-thienopyridine derivatives) and thrombin receptor-activating peptide 6 (TRAPtest, designed to evaluate the effect of GPIIbIIIa receptor and protease-activated receptor inhibitors). Based on previously defined cutoff values, ADP values < 22 U, ASPI values < 30 U and TRAP values < 75 U were considered pathologic. [12,16] All multiplate analyses were performed prior to surgery.

### Clinical parameters

Demographic, clinical and laboratory data were extracted from the patient database. Use of antiplatelet drugs were documented if self-reported by patient upon admission, available within the previous therapy plan or administered through emergency physicians prior to arrival.

### Outcome variables

Primary endpoints were the amount of intraoperative or total transfused blood products; namely Red cell concentrates (RCC, 350 ml unit), Platelet concentrates (PC, 250 ml unit) and Fresh frozen plasma (FFP, 250 ml unit). All blood products transfused beginning from anesthesia induction to transfer of the patient to intensive care were considered as intraoperative transfused products. All products transfused from arrival of the patient to discharge were considered in the total transfused products. Secondary endpoints were surgical re-exploration due to bleeding and 30-day all-cause mortality. Each surgical re-exploration was further analyzed to determine the indication. Only those with bleeding as the main indication were included as secondary endpoint.

### Statistical analysis

All continuous data are presented as mean ± standard deviation or as median and interquartile ranges (25% and 75%) in the case of non-normally distributed data. Categorical variables are presented as numbers and percentages. The distribution of data was assessed by the Shapiro-Wilk test. Comparisons between groups were conducted using a two-sided t-test or Mann-Whitney U test for continuous variables and the chi-square test or Fisher’s exact test for categorical variables, depending on the data distribution. A *p*-value < 0.05 was considered statistically significant. Statistical analysis was done using SPSS (IBM Corp. Released 2021. IBM SPSS Statistics for Macintosh, Version 28.0. Armonk, NY: IBM Corp).

## Results

### Demographic and clinical data

A total of 188 patients presented with ATAAD between 2019–2021. Iatrogenic cases (n = 8) were excluded. Patients were predominantly male (68.3%) and had a median age of 64 (IQR: 57–77.5) years. The demographic and clinical parameters are summarized in Table 1.

**Table 1:** Demographics, clinical data and laboratory parameters of ATAAD patients.

Variable	*n* = 180
Age, (years)	64 (57–77.5)
Female, *n (%)*	57 (31.7%)
BMI, kg/m^2^	27.1 (24.6–31)
Self-reported antiplatelet use	
Aspirin	35 (19.3%)
Clopidogrel	7 (3.9%)
DAPT	5 (2.8%)
Acute aortic syndrome, *n* (%)	
Acute aortic dissection	168 (93.3%)
Ruptured Aneurysm	7 (3.9%)
PAU	2 (1.1%)
Intramural Hematoma	3 (1.7%)
Hemoglobin (g/dL)	12.6 ± 1.8
Platelet count (/mL)	193.5 (155.2–232.5)
aPTT (r)	1 (0.9–1.2)
INR	1.2 (1.1–1.3)
PT (sec)	14 (13.2–15.6)
Multiplate tests with abnormal aggregation response	
ADP, *n* (%)	47 (26%)
ASPI, *n* (%)	89 (49.2%)
TRAP, *n* (%)	113 (63.5%)
Intraoperative transfused blood products	
RCC, units	2.4 ± 3.4
PC, units	3.4 ± 2.6
FFP, units	5.6 ± 6
Total transfused blood products	
RCC, units	6.7 ± 8.1
PC, units	4.8 ± 3.3
FFP, units	8.4 ± 8.1
Operation time (min)	358 (281–450)
Reperfusion time (min)	63 (35–94)
Cardiopulmonary bypass time (min)	190 (146–242)
Operation temperature (°C)	28.2 ± 3.9
Aortic valve surgery, *n* (%)	111 (61.3%)
Sinus involvement, *n* (%)	115 (63.9%)
Reimplantation of coronary arteries, *n* (%)	39 (21.5%)
Aortic vessel operation location, *n* (%)	
Root	131 (72.8%)
Ascending aorta	158 (87.8%)
Hemiarch	107 (59.4%)
Arch	32 (17.8%)
Descending thoracic aorta	4 (2.2%)
Surgical exploration for Bleeding, *n* (%)	8 (4.4%)
Surgical exploration for Tamponade, *n* (%)	18 (9.9%)
Surgical evacuation for Hemothorax, *n* (%)	2 (1.1%)
Intraoperative death, *n* (%)	6 (3.3%)
Hospital length of stay (days)	12 (7–17)
30-day all-cause mortality, *n* (%)	33 (18.3%)

Data are presented as median (interquartile range)/mean ± SD and n (%) for categorical variables. DAPT – Dual antiplatelet therapy, BMI – Body mass index, PAU – Penetrating aortic ulcer, CABG – Coronary artery bypass graft, ADP – Adenosine diphosphate, ASPI – Arachidonic acid, TRAP – Thrombin-receptor-activated peptide 6, aPTT – Activated partial thromboplastin time, INR – International normalized ratio, RCC – Red cell concentrates, PC – Platelet cell concentrates, FFP – Fresh frozen plasma, PT – Prothrombin time

### Antiplatelet use and coagulation parameters

Forty-seven patients (26.1%) reported antiplatelet use. Among them, 35 patients (19.3%) reported aspirin use, 7 patients (3.9%) reported clopidogrel use, and 5 patients (2.8%) were on dual antiplatelet therapy (DAPT). A total of 39 patients (21.6%) displayed abnormal aggregation responses to one activator, while 69 (38.1%) had abnormal response to two activators, and 36 (20%) showed abnormal aggregations responses across all three activators. Preoperative laboratory parameters and platelet function assessed using Multiplate are depicted in **Table 1**.

### Clinical outcomes

No significant difference was seen in age, sex, standard anticoagulation tests, platelet counts and self-reported use of anti-platelet drugs. No significant differences in operation parameters except for a longer cross-clamp time in patients with abnormal aggregation response against TRAP (106 vs 90.5 minutes, p = 0.047) were observed. There were also no significant differences in surgical re-exploration for bleeding and 30-day all-cause mortality. For a detailed comparison of variables see Supporting information in S1–S4 Files.

**Fig 1:**
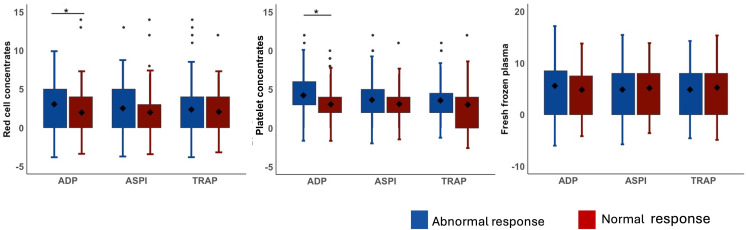
Intraoperative transfused blood products. ADP – Adenosine diphosphate, ASPI – Arachidonic acid, TRAP – Thrombin-receptor-activated peptide 6, * - p < 0.05.

No significant differences in intraoperative or total blood products transfused in patients with abnormal aggregation responses to ASPI and TRAP. However, patients with abnormal ADP tests had a higher intraoperative need for RCC (3.4 ± 3.9 versus 2.1 ± 3.2, *p* = 0.039) and PC (4.2 ± 2.9 versus 3.1 ± 2.4, *p* = 0.015). (See Fig 1). Patients with abnormal ADP responses also had a higher total need for PC (8.1 ± 7.8 versus 6.2 ± 8.1, p = 0.008).

## Discussion

To the best of our knowledge, this is the first study to explore the use of multiplate aggregometry in ATAAD patients. Our study reveals the incidence of platelet dysfunction demonstrated by abnormal aggregation responses and the associated higher need for transfusion of blood products. Furthermore, standard anticoagulation tests and self-reported use of DAPT did not show significant differences, emphasizing the potential of MPA to detect platelet dysfunctions that conventional tests may miss.

MPA has been widely investigated in patients with coronary bypass surgery. [17–19] These studies have highlighted the greater significance of MPA compared to routine anticoagulation tests in predicting bleeding complications, leading to consensus recommendations in cardiac surgery patients. [19] A prospective randomized clinical trial in 100 cardiac surgery patients has even shown that POC testing using MPA reduced the need for perioperative exposure to allogenic blood produced and improved clinical outcomes. [14] Our study demonstrates the significance of aggregation response testing in ATAAD patients. In line with a previous study [20], our study also showed that MPA was a better indicator of platelet dysfunction and need for platelet transfusion than self-reported DAPT use. This is even more crucial in ATAAD patients, where emergency surgical repair is necessary, and a reliable history-taking and preoperative optimization of hemostasis is not always possible.

Our study revealed that ATAAD patients with abnormal ADP aggregation response required higher number of blood products. These findings are consistent with previous studies [21–25] that have reported an association between abnormal ADP response and increased need for platelet transfusions.

We observed no difference in clinical complications such as incidences of surgical re-exploration and 30-day-all-casue mortality. This is also in line with recent studies in cardiac [26] and non-cardiac surgery patients [27], that showed that timely and adequate treatment of postoperative bleeding does not necessarily correlate with higher mortality rates.

This study has several limitations. The retrospective and monocentric nature of the study are worth mentioning. Cut-off values for abnormal aggregation responses were extrapolated from previous studies in other cardiac surgery patients. Prospective studies with ATAAD patients and determination of an optimal cut off value can help in accurately identifying the patients at risk for bleeding. In our study, correction of aggreagation response for platelet count was not done. MPA is also proven to be sensitive to other intraoperative factors such as hypothermia and effects of CPB. [28] No immediate postoperative MPA measurement was done to account for these factors. Bleeding in cardiac surgery patients is multifactorial. This study only analyzed platelet dysfunction without consideration of the other components of hemostasis, factor deficiencies, or perioperatively administered anticoagulation medications. Furthermore, bleeding was not quantified but the amount of transfused blood products is used as a surrogate. In addition, the indication for transfusion was at the discretion of the treating physician. A multicenter study could provide a bigger cohort of ATAAD patients and eventually shine some light on the different clinical practices of transfusions.

In conclusion, this study demonstrated that MPA can be used to assess platelet dysfunction in ATAAD patients and its routine use as a preoperative POC tool should be encouraged. Identification of patients with higher transfusion needs can help surgical teams stratify patients and better tailor blood product transfusions.

## Supporting information

S1 File Comparison of primary outcomes. (DOCX)

S2 File Clinical outcomes depending on ASPI test results. (DOCX)

S3 File Comparison of preoperative, intraoperative, and postoperative variables between groups with ADP tests. (DOCX)

S4 File Comparison of preoperative, intraoperative, and postoperative variables between groups with TRAP tests. (DOCX)

## Abbreviations

ADP: Adenosine diphosphate
aPTT: Activated partial thromboplastin time
ASPI: Arachidonic acid
ATAAD: Acute type A aortic dissection
BMI: Body mass index
CABG: Coronary artery bypass graft
DAPT: Dual antiplatelet therapy
FFP: Fresh frozen plasma
GPIIb/IIIa: Glycoprotein IIb/IIIa
INR: International normalized ratio
IQR: Interquartile ratio
MPA: Multiplate aggregometry
PAU: Penetrating aortic ulcer
PC: Platelet concentrates
POC: Point-of-care
PT: Prothrombin time
RCC: Red cell concentrates
SPSS: Statistical Package for the Social Sciences
TRAP: Thrombin-receptor-activated peptide 6
