# Supplementary material for: Assessing transfusion need in patients with type A aortic dissection with multiplate aggregometry
Source: PLoS One. 2025 Jul 17;20(7):e0324477. doi: 10.1371/journal.pone.0324477 (PMC12270149; doi:10.1371/journal.pone.0324477)
Supplement: S1 File — (DOCX) [file pone.0324477.s001.docx]

**S1: Comparison of primary outcomes**

|  | **ADP test** | | | **ASPI test** | | | **TRAP test** | | | |
| --- | --- | --- | --- | --- | --- | --- | --- | --- | --- | --- |
| **Variables** | **Abnormal (n=47)** | **Normal (n=131)** | ***P*** | **Abnormal (n=89)** | **Normal (n=91)** | ***P*** | **Abnormal (n=115)** | **Normal (n=65)** | ***P*** | |
| **Intraoperative transfused blood products** | | | |  |  |  |  |  |  | |
| RCC, units | 3.4 ± 3.9 | 2.1 ± 3.2 | ***0.039*** | 2.9 ± 3.9 | 1.9 ± 2.7 | *0.224* | 2.6 ± 3.8 | 2.1 ± 2.6 | *0.602* | |
| PC, units | 4.2 ± 2.9 | 3.1 ± 2.4 | ***0.015*** | 3.6 ± 2.8 | 3.1 ± 2.3 | *0.266* | 3.6 ± 2.4 | 3 ± 2.8 | *0.088* | |
| FFP, units | 7.4 ± 8.2 | 5.1 ± 4.9 | *0.252* | 5.6 ± 6.6 | 5.7 ± 5.4 | *0.391* | 5.9 ± 6.5 | 5.2 ± 5.1 | *0.847* | |
| **Total transfused blood products** | | | |  |  |  |  |  |  | |
| RCC, units | 8.1 ± 7.8 | 6.2 ± 8.1 | 0.087 | 7.8 ± 9.2 | 5.6 ± 6.7 | *0.182* | 7 ± 8.3 | 6.2 ± 7.6 | *0.760* | |
| PC, units | 5.7 ± 3.1 | 4.5 ± 3.4 | **0.008** | 5.3 ± 3.7 | 4.4 ± 2.9 | *0.079* | 5 ± 3.4 | 4.5 ± 3.2 | *0.457* | |
| FFP, units | 10.7 ± 9.4 | 7.6 ± .7.5 | 0.054 | 9.2 ± 9.5 | 7.7 ± 6.4 | *0.738* | 8.9 ± 8.9 | 7.6 ± 6.4 | *0.754* | |
| RCC – Red cell concentrates, PC – Platelet concentrates, FFP – Fresh frozen plasma | | | | | | | | | |  |
