# Supplementary material for: Assessing transfusion need in patients with type A aortic dissection with multiplate aggregometry
Source: PLoS One. 2025 Jul 17;20(7):e0324477. doi: 10.1371/journal.pone.0324477 (PMC12270149; doi:10.1371/journal.pone.0324477)
Supplement: S2 File — (DOCX) [file pone.0324477.s002.docx]

**S2: Clinical outcomes depending on ASPI test results**

|  | **ASPI test** | |  |
| --- | --- | --- | --- |
| **Variables** | **Abnormal (n=89)** | **Normal (n=91)** | ***P*** |
| Age, (years) | 67 (57.7 – 78) | 62 (56 – 75) | 0.448 |
| Female, n(%) | 32 (17.8%) | 25 (13.9%) | 0.221 |
| Anticoagulant use |  |  |  |
| Aspirin | 19 (10.6%) | 16 (8.8%) | 0.523 |
| Clopidogrel | 5 (2.8%) | 2 (1.1%) | 0.235 |
| DAPT | 4 (2.2%) | 1 (0.6%) | 0.166 |
| INR | 1.12 (1.06 – 1.22) | 1.2 (1.1 – 1.3) | 0.061 |
| PT (sec) | 13.4 (12.7 – 14.6) | 14.4 (13.2 – 16) | 0.060 |
| aPTT (r) | 0.98 (0.9 – 1.16) | 1 (0.91 – 1.16) | 0.858 |
| Platelet count (/mL) | 196 (146 – 230) | 185.5 (159.2 – 236.8) | 0.369 |
| Intraoperative variables | | | |
| CPB (min) | 193 (146 – 258.8) | 186.5 (141.8 – 230) | 0.388 |
| Operation time (min) | 364 (283.8 – 455) | 333 (275.2 – 449.5) | 0.494 |
| Operation temperature (°C) | 28 (26 – 28.4) | 28 (26.9 – 29.2) | 0.418 |
| Cross-clamp time (min) | 92.5 (67 – 119) | 90.5 (68 – 128.5) | 0.510 |
| Postoperative variables, n(%) |  |  |  |
| Surgical exploration for bleeding | 5 (2.8%) | 3 (1.7%) | 0.450 |
| Surgical exploration for Tamponade | 11 (6.1%) | 7 (3.9%) | 0.297 |
| Surgical exploration for Hemothorax | 0 (0%) | 2 (1.1%) | 0.160 |
| Length of hospital stay, (days) | 11 (6 – 17) | 12 (6 – 16.8) | 0.972 |
| 30-day all-cause mortality | 25 (13.9%) | 18 (10%) | 0.191 |
| ASPI – Arachidonic acid, aPTT – Activated partial thromboplastin time, INR – International normalized ratio, CPB - Cardiopulmonary bypass time, DAPT – Dual antiplatelet therapy, PPSB – Human prothrombin complex concentrate | | | |
