# Supplementary material for: Assessing transfusion need in patients with type A aortic dissection with multiplate aggregometry
Source: PLoS One. 2025 Jul 17;20(7):e0324477. doi: 10.1371/journal.pone.0324477 (PMC12270149; doi:10.1371/journal.pone.0324477)
Supplement: S3 File — (DOCX) [file pone.0324477.s003.docx]

**S3: Comparison of preoperative, intraoperative, and postoperative variables between groups with ADP tests**

|  | **ADP test** | |  | |  |  |
| --- | --- | --- | --- | --- | --- | --- |
| **Variables** | **Abnormal (n=47)** | **Normal (n=131)** | ***P*** | |  |  |
| Age, (years) | 64 (58 – 75.5) | 62 (53 – 74) | 0.691 | |  |  |
| Female | 13 (7.2%) | 44 (24.4%) | 0.455 | |  |  |
| Anticoagulant use |  |  |  | |  |  |
| Aspirin | 9 (5%) | 26 (14.4%) | 0.918 | |  |  |
| Clopidogrel | 3 (1.7%) | 4 (2.2%) | 0.314 | |  |  |
| DAPT | 2 (1.1%) | 3 (1.7%) | 0.484 | |  |  |
| INR | 1.2 (0.92 – 1.29) | 1.1 (0.9 – 1.15) | 0.821 | |  |  |
| PT (sec) | 13.9 (13.2 – 14.5) | 14.2 (13 – 15.6) | | 0.811 | | |
| aPTT (r) | 1 (32.4 – 45.8) | 0.99 (31.7 – 40.3) | 0.962 | |  |  |
| Platelet count (G/L) | 174 (138.8 – 213.5) | 196 (162 – 239.5) | 0.097 | |  |  |
| Intraoperative variables |  |  |  | |  |  |
| CPB (min) | 206 (141 – 264.2) | 190 (149 – 239) | 0.672 | |  |  |
| Operation time | 394.5 (281.2 – 529) | 353 (283.5 – 420) | 0.094 | |  |  |
| Operation temperature | 28 (26 – 29) | 28 (26.9 – 28) | 0.910 | |  |  |
| Cross-clamp time | 98 (72.8 – 129.5) | 91 (67 – 126.5) | 0.736 | |  |  |
| Postoperative variables |  |  |  | |  |  |
| Surgical exploration for bleeding | 3 (1.7%) | 5 (2.8%) | 0.466 | |  |  |
| Surgical exploration for Tamponade | 4 (2.2%) | 13 (7.2%) | 0.777 | |  |  |
| Surgical exploration for Hemothorax | 1 (0.6%) | 1 (0.6%) | 0.447 | |  |  |
| Length of hospital stay, (days) | 10 (6 – 13) | 12 (6 – 18.5) | | 0.249 | |  |
| 30-day all-cause mortality | 9 (5%) | 22 (12.2%) | 0.715 | |  |  |
| ADP – Adenosine diphosphate, aPTT – Activated partial thromboplastin time, INR – International normalized ratio, CPB - Cardiopulmonary bypass time, DAPT – Dual antiplatelet therapy, PPSB – Human prothrombin complex concentrate | | | | |  |  |
