# Supplementary material for: Assessing transfusion need in patients with type A aortic dissection with multiplate aggregometry
Source: PLoS One. 2025 Jul 17;20(7):e0324477. doi: 10.1371/journal.pone.0324477 (PMC12270149; doi:10.1371/journal.pone.0324477)
Supplement: S4 File — (DOCX) [file pone.0324477.s004.docx]

**S4: Comparison of preoperative, intraoperative, and postoperative variables between groups with TRAP tests**

|  | **TRAP test** | | | | | | |  | | |
| --- | --- | --- | --- | --- | --- | --- | --- | --- | --- | --- |
| **Variables** | **Abnormal (n=115)** | | | | **Normal (n=65)** | | | ***P*** | | |
| Age, (years) | 62.5 (56.2 – 72.8) | | | | 65 (54.8 – 79) | | | 0.352 | | |
| Female | 36 (20%) | | | | 21 (11.7%) | | | 0.889 | | |
| Anticoagulant use |  | | | |  | | |  | | |
| Aspirin | 23 (12.8%) | | | | 12 (6.7%) | | | 0.802 | | |
| Clopidogrel | 6 (3.3%) | | | | 1 (0.5%) | | | 0.220 | | |
| DAPT | 4 (2.2%) | | | | 1 (0.5%) | | | 0.447 | | |
| INR | 1.15 (1.1 -1.3) | | | | 1.15 (1.04 – 1.3) | | | 0.703 | | |
| PT (sec) | | 14 (13.2 – 15.6) | | 13.9 (12.6 – 15.4) | | 0.702 | | |  |  |
| aPTT (r) | 0.99 (0.91 – 1.15) | | | | 1 (0.87 – 1.16) | | | 0.296 | | |
| Platelet count (G/L) | 185 (152.2 – 225.5) | | | | 199.5 (171.5 – 243.2) | | | 0.276 | | |
| Intraoperative variables |  | | | |  | | |  | | |
| CPB (min) | 197 (144.5 – 260.2) | | | | 190 (149.2 – 220.2) | | | 0.424 | | |
| Operation time | 374.5 (286.2 – 493.2) | | | | 334.5 (277.5 – 418.2) | | | 0.126 | | |
| Operation temperature | 28 (26.8 – 28.6) | | | | 28 (26.2 – 28) | | | 0.675 | | |
| Cross-clamp time | 98 (73.2 – 137) | | | | 86.5 (62.2 – 115.2) | | | **0.047** | | |
| Postoperative variables |  | | | |  | | |  | | |
| Surgical exploration for bleeding | 2 (1.1%) | | | | 6 (3.3%) | | | 0.099 | | |
| Surgical exploration for Tamponade | 14 (7.8%) | | | | 4 (2.2%) | | | 0.196 | | |
| Surgical exploration for Hemothorax | 1 (0.5%) | | | | 1 (0.5%) | | | 0.681 | | |
| Length of hospital stay, (days) | | | 10 (6 – 16) | | 12 (6 – 18.2) | | 0.809 | | |  |
| 30-day all-cause mortality | 23 (20%) | | | | 10 (15.4%) | | | 0.442 | | |
| TRAP – Thrombin-receptor-activated peptide 6, aPTT – Activated partial thromboplastin time, INR – International normalized ratio, CPB - Cardiopulmonary bypass time, DAPT – Dual antiplatelet therapy, PPSB – Human prothrombin complex concentrate | | | | | | | | | | |
